# Polyether-Thiourea-Siloxane Copolymer Based on H-Bonding Interaction for Marine Antifouling

**DOI:** 10.3390/molecules28083574

**Published:** 2023-04-19

**Authors:** Mengyu Li, Liyang Nan, Boxuan Zhang, Junjun Kong, Yufeng Wang, Miao Ba

**Affiliations:** Department of Material Science and Engineering, Changshu Institute of Technology, Changshu 215500, China

**Keywords:** marine antifouling, copolymer, H-bonding interaction, leaching, fouling release

## Abstract

By introducing thiourea and ether groups into MQ silicone resin polymer via free radical polymerization, a polyether-thiourea-siloxane (PTS) copolymer was synthesized. The characterization of the synthesized copolymer indicated the occurrence of H-bonding interactions and a narrow molecular weight polydispersity index. Antifouling coatings were produced by incorporating the synthesized copolymer and phenylmethylsilicone oil (PSO). The addition of a minute amount of copolymer enhanced the hydrophobicity of the coating by increasing its surface roughness. However, excessive addition of copolymer resulted in a significant deterioration of the coating surface smoothness. The copolymer improved the mechanical properties of the coating, but excessive addition decreased the crosslinking density and weakened the mechanical performance. With increasing copolymer addition, the leaching of PSO was significantly improved due to the change in the storage form of PSO in the coating caused by the copolymer. Based on the H-bonding interaction of the copolymer, the adhesion strength between the coating and the substrate was significantly improved. However, excessive addition of copolymer did not infinitely enhance the adhesion strength. The antifouling performance demonstrated that an appropriate amount of copolymer could obtain adequate PSO leaching efficiency, thereby effectively enhancing the antifouling performance of the coating. In this study, the prepared coating P_12_ (12 g of PTS in 100 g of PDMS) showed the most effective antifouling performance.

## 1. Introduction

Biofouling is the accumulation of living organisms on the surfaces of submerged objects in the marine environment [1,2]. The bacteria, algae, barnacles, and mollusks that adhere to these surfaces are capable of forming biofilms, which are complex and highly organized communities [3]. Biofouling can develop on numerous surfaces, such as ships, offshore oil rigs, and other man-made structures. This process can result in a variety of undesirable outcomes. It can increase a ship’s drag and weight, resulting in an increase in fuel consumption and emissions [4]. Biofouling can also cause structural damage to marine vessels, offshore structures, and other submerged surfaces. Additionally, biofouling can have a negative effect on marine ecosystems by altering the distribution and abundance of species and reducing the amount of available light for photosynthetic organisms [5,6]. Biofouling can also serve as a vector for the introduction and dissemination of invasive species, resulting in additional disturbances to marine ecosystems. Therefore, the control and prevention of marine biofouling are essential for maintaining the safety and health of marine environments and structures.

Several methods exist for controlling and preventing marine biofouling [7]. The most common method is the application of antifouling coatings to submerged surfaces. Traditional marine antifouling coatings contain toxic compounds that prohibit marine organism attachment [8]. However, these coatings can have detrimental effects on marine ecosystems and contribute to the release of harmful chemicals into the water. In recent years, there has been a growing interest in the use of non-toxic or eco-friendly methods to control and prevent marine biofouling [9]. Compared with coatings containing toxic agents, environmentally friendly antifouling coatings can effectively inhibit the adhesion behavior of marine fouling organisms via physical or biomimetic means, or by using environmentally friendly bactericides, without negatively impacting the marine ecological environment [10,11]. Fouling-resistant, fouling-release (FR), and fouling-degrading antifouling coatings make up the majority of eco-friendly antifouling coatings currently available.

Polydimethylsiloxane (PDMS)-based polymers have been extensively investigated and applied in the development of fouling release coatings due to their unique properties [7,12]. These properties include low surface energy, high hydrophobicity, and a low elastic modulus. The low surface energy of PDMS-based polymers makes it difficult for marine organisms to attach to the surface, which reduces fouling [13]. The high hydrophobicity of PDMS-based polymers reduces the adhesion of marine organisms, and also facilitates their easy removal from surfaces. In addition, the lower elastic modulus of the coating surface causes adherent fouling organisms to disengage via a low-energy peeling mechanism, effectively enhancing the coating’s fouling release capability [14,15,16]. In the field of fouling release, PDMS-based antifouling coatings are therefore a focal point of research.

However, the commercial application of PDMS-based FR coatings faces pressing challenges as well. The most significant flaw is that, as a non-polar material, PDMS polymer has weak adhesion to various polar substrates, making it difficult for PDMS coatings to stably adhere to metal substrates in a stable manner over time [17,18,19,20,21]. Once the coating is subjected to impact or friction, it will flake off in large quantities, exposing the metal ocean facility to a hazardous environment. Measures taken to enhance the adhesion strength of PDMS-based coatings, such as chemical modification and micro- or nanoparticle modification, may also adversely affect the low surface properties or elasticity of the coating [3,15,22,23], thereby diminishing its antifouling effect. Other detrimental factors include inadequate wear resistance, which reduces its anti-fouling properties when subjected to mechanical wear and tear, and the inability to fully reflect its fouling release characteristics on low-speed ships or stationary equipment (due to a lack of sufficient seawater flow) [24,25].

On the premise of the development of PDMS-based FR coatings, non-reactive silicone oil was incorporated into the coating to mimic the mucous secretion behavior of marine animals’ skin surfaces and enhance the fouling release impact of the coating [7,8,18,19,22]. The more prominent advantage is that the removal rate of fouling from the original coating is affected by the elastic modulus of the coating, and a reasonable silicone oil release efficiency can overcome the increased difficulty of removing adherent fouling organisms due to the enhancement of the elastic modulus [8,19]. This can provide a remedy for the deterioration of the pertinent properties of modified PDMS-based FR coatings.

**Figure 1:**
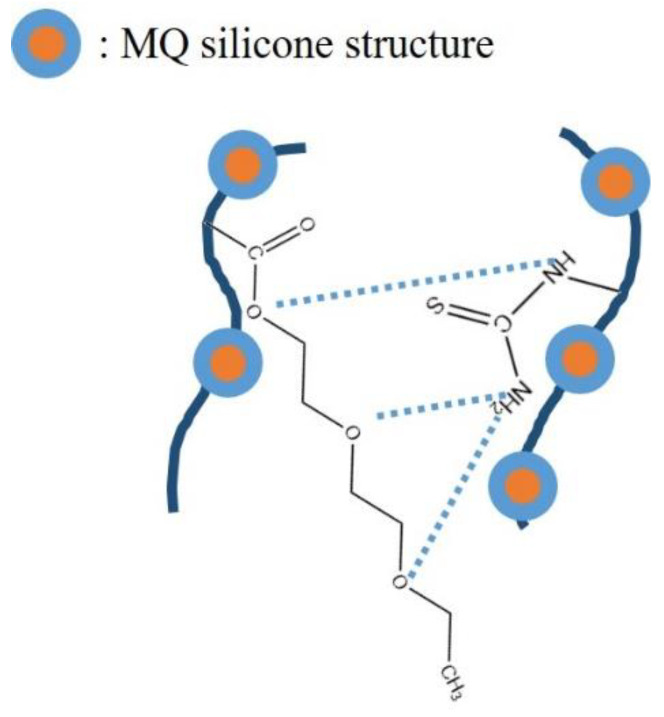
Synthesis of the polymer PTS.

In this work, a modified MQ silicone copolymer (PTS) with H-bonding interactions was synthesized based on the free radical copolymerization reaction, which produced a random copolymer. On the basis of the polymer solution theory, this copolymer was compounded with PDMS resin and phenyl silicone oil (PSO) before being used to create coatings. As additives, PTS and PSO exist in the spaces of the cross-linked PDMS network structure. The principal film-forming material of the entire cured coating is still cross-linked PDMS elastomer. Phase separation is attained due to the difference in molecular structure between the additives and the PDMS elastomer, and PSO can precipitate from the coating surface. PTS has exceptional polarity properties as a result of H-bonding interactions between ether and thiourea groups; as a result, it does not exist uniformly in the PDMS elastomer but is instead enriched and segregated. In addition, PTS exhibits outstanding adhesion due to its molecular structural properties, thereby enhancing the bonding strength of the blended coating to the substrate. In addition, the addition of a minor quantity of additive does not diminish the antifouling performance of the PDMS coating. Modifications in the PSO’s leaching behavior further improve the coating’s antifouling properties. The characteristic structure of the synthetic copolymer (PTS) is shown in Figure 1.

## 2. Results and Discussion

### 2.1. Synthesis of PTS

The interaction between thiourea and ether groups results in a strong H-bonding effect [26], which can effectively enhance the adhesion to various substrates. In this study, two monomers, ATU and EOEOEA, were used to modify VMQ resin, thereby introducing corresponding functional groups into the modified polysiloxane polymer. The three reactants can be solubilized without solvent at reasonable molar ratios, and the synthesis ratio was based on the solubility involved. After multiple extractions and purifications, the dried copolymer was analyzed with GPC equipment, and the detailed results are shown in Supplementary File S1. Within the accurate detection range of the GPC equipment, only one peak appeared, among which Mn = 120,718 and Mw = 200,874. Furthermore, the calculated polydispersity index (Mw/Mn) was 1.66, revealing a narrow molecular weight distribution. A polydispersity index less than 2.00 indicated that the synthesized copolymer had a more concentrated molecular weight, which was beneficial for analyzing the performance of the prepared coating. Meanwhile, the existence of a single peak in the GPC spectra also demonstrated that the purified polymer contained no reactants.

**Figure 2:**
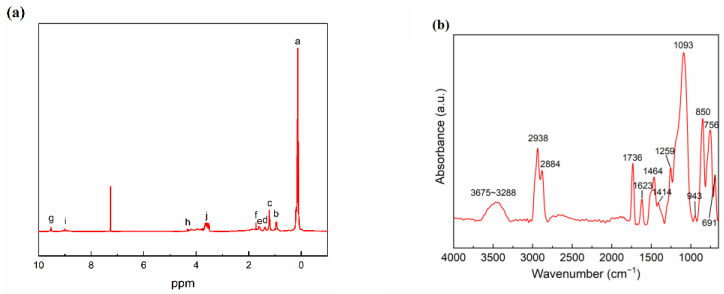
(**a**) the ^1^H-NMR spectra of the copolymer; (**b**) the FT-IR spectra of the copolymer.

The molecular structure and characteristics of the copolymer were further analyzed using ^1^H-NMR spectra (Figure 2a), and a detailed description of the ^1^H-NMR chemical shifts is provided in Supplementary File S2, and the ^1^H-NMR chemical shifts of VMQ are shown in Supplementary File S3. The incorporation of thiourea groups into the polymer chain was confirmed by the detection of characteristic signatures of the amino and imino protons from ATU, such as –(C=S)–NH_2_ at 9.53 ppm (Hg) and –NH–(C=S)– at 9.01 ppm (Hi). Due to the minute amount of ATU used as a reactant, the corresponding proton signals of the thiourea groups in the polymer were of low intensity and small area. Similarly, characteristic signals of the ether methylene protons (–CH_2_–) from EOEOEA were detected in the range of 3.52–3.63 ppm (Hj), with a broad chemical shift peak determined by the proton characteristics of the ether methylene. In addition, characteristic signals of the ester methylene protons from EOEOEA (Hh, 4.32 ppm, –CO–O–CH_2_) were also detected, with a smaller chemical shift area compared to the ether methylene protons. These tests confirmed the successful incorporation of thiourea and ether groups in the synthesized copolymer, while no signals of the olefinic protons of the three reactants (ATU at 5.06–5.84 ppm, EOEOEA at 5.83–6.12 ppm, and VMQ at 2.18 ppm) were detected, indicating that the copolymer was synthesized via free radical copolymerization and that the unreacted reactants were completely removed by extraction.

Moreover, by analyzing the FT-IR characteristic absorption spectrum of the copolymer (Figure 2b), it was evident that, due to the strong H-bonding interactions, the copolymer exhibited a prominent hydroxyl feature absorption peak at 3675–3288 cm^−1^ with a broad peak width. The stretching vibration peaks of methyl (–CH_3_) and methylene (–CH_2_–) were observed at 2938 cm^−1^ and 2884 cm^−1^, respectively, with the characteristic peak of methyl being stronger than that of methylene, which was due to the presence of a large number of methyl groups in the VMQ copolymer. The primary structural unit of the synthesized copolymer is VMQ. The stretching vibration peak of the ester carboxyl group R–COO–R was detected at 1736 cm^−1^, the deformation bending vibration peak of the –N–H bond was detected at 1623 cm^−1^, and the bending vibration peak of the –N–C=S of the thiourea group was observed at 943 cm^−1^. However, the stretching vibration peak of the –Si–CH_3_ group from the VMQ resin at 1259 cm^−1^ obscured the anti-symmetric stretching vibration peak of the –C-O– group around 1278 cm^−1^, preventing direct observation of the ether group in the FT-IR spectrum of the copolymer. Importantly, there were no unsaturated olefinic groups in the spectra of the copolymer (at 3060 cm^−1^, 3016 cm^−1^, 1633 cm^−1^, 1598 cm^−1^, and 984 cm^−1^), which confirmed the completeness of the free radical copolymerization process (Supplementary File S4).

### 2.2. Surface Properties of the Prepared Coatings

**Figure 3:**
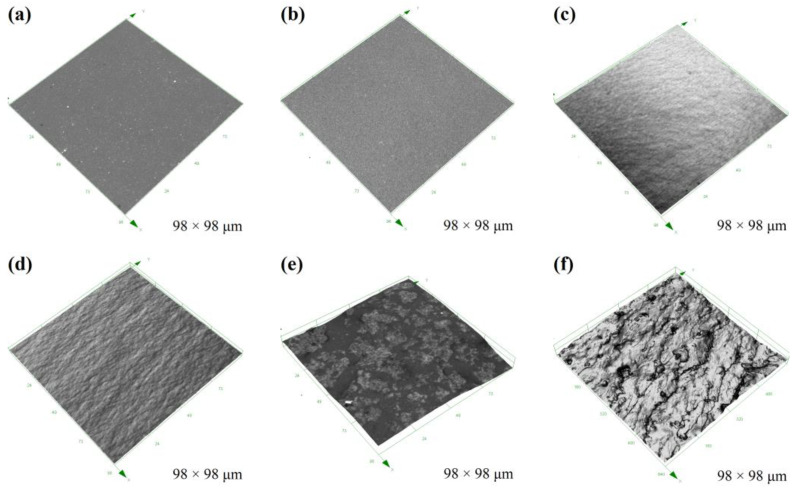
The surface microstructure of the prepared coatings by CLSM. For the P20 sample, the field of view is 640 × 640 µm, while the field of view for other samples is 98 × 98 µm. (**a**) P_0_; (**b**) P_4_; (**c**) P_8_; (**d**) P_12_; (**e**) P_16_; (**f**) P_20_.

**Figure 4:**
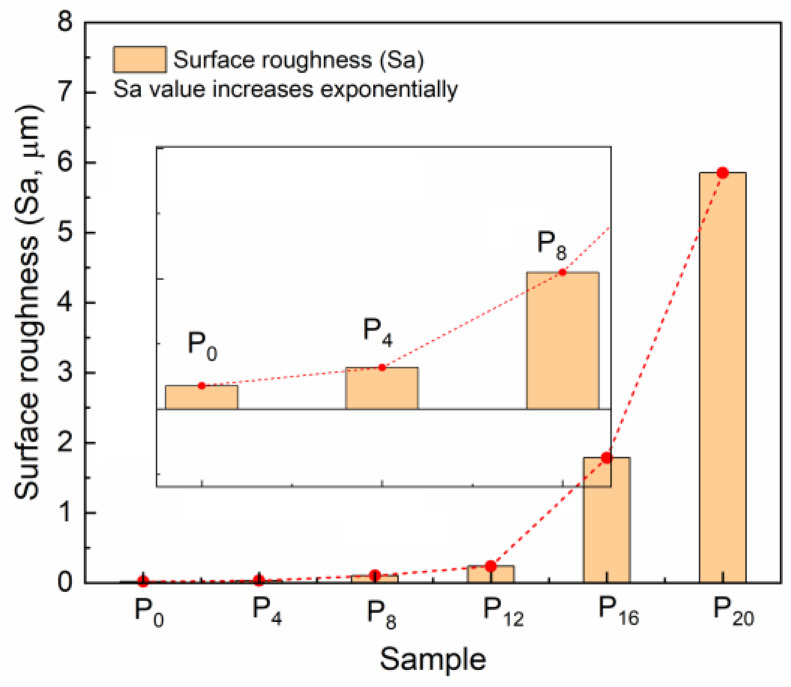
The surface roughness values of the prepared coatings.

A series of FR coatings were prepared based on synthesized copolymers. The hydrophobicity of the coatings was evaluated by water contact angle and calculated surface free energy (Table 1). As a control sample (P_0_), the coatings exhibited excellent hydrophobicity and low surface free energy. With the addition of PTS to the FR coatings, the water contact angle of the prepared coatings initially increased. However, the hydrophobicity of the prepared coatings deteriorated rapidly and ultimately became hydrophilic (<90°) when the amount of copolymer added to the 100 g of PDMS resin exceeded 12 g. Likewise, the surface free energy of the hydrophilic coatings also increased sharply. To explore the changes in surface properties of the coatings, the surface microstructure of the prepared coatings was observed using CLSM (Figure 3). The results showed that when the amount of copolymer added was small (e.g., P_4_), the surface of the coating exhibited similar smoothness as the control coating. Within the range of copolymer additions from 4 g to 12 g, the surface smoothness of the prepared coatings degraded progressively, but the overall microstructure exhibited uniform undulations. For hydrophobic coatings, the increase in micro-roughness can, to some extent, enhance the hydrophobicity of the coatings. However, excessive copolymer addition resulted in a significant deterioration of the surface morphology of the prepared coatings, and the excessively rough surface structure ultimately led to a transition to hydrophilic coatings. The surface roughness results (Figure 4) measured by CLSM also confirmed the surface changes. With the addition of copolymers, the values of surface roughness of the coating exhibit an exponential function relationship. Extremely high values of the surface roughness inevitably imply severe damage to the flatness of the coating. The transition from hydrophobic to hydrophilic appeared to hinder the anti-fouling performance of the coatings.

**Figure 5:**
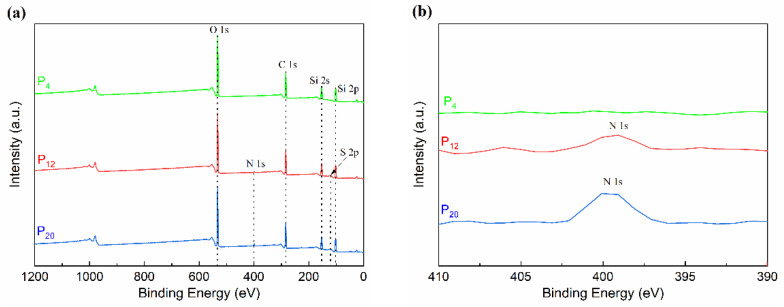
(**a**) XPS spectra; (**b**) locally amplified spectra.

Furthermore, the elemental composition of the coating surface region was analyzed using XPS (Figure 5). N and S elements were detected on the surface of the coating with the addition of the copolymer, indicating the presence of the copolymer in the coating surface area. During the three-dimensional crosslinking process of the organosilicon elastomer, the copolymer exists in a state of freedom within the three-dimensional network space. Due to the strong hydrogen bond interaction of the copolymer, it tends to accumulate in the coating-substrate region (interface region). In the stage where a small amount of copolymer was added, it was difficult to detect N and S elements on the prepared coating surface (e.g., P_4_). With the increasing copolymer addition, the interface region was unable to store additional free copolymers; consequently, free copolymers also appeared in the surface region, and the presence of copolymers disrupted the coating surface’s uniformity. Due to the difference in molecular structure between the non-polar PDMS elastomer and the copolymer, they interacted with each other, causing wrinkles or steps on the coating surface and thereby influencing the hydrophobicity and surface free energy of the coating.

### 2.3. The Mechanical Properties of the Prepared Coatings

**Figure 6:**
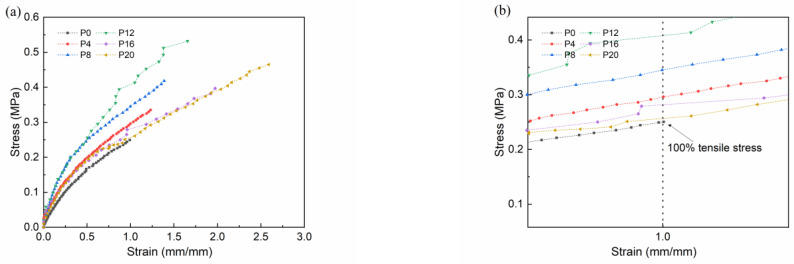
(**a**) The stress-strain curve of the prepared coatings; (**b**) local amplification.

The mechanical properties of the prepared coatings with the incorporation of synthetic copolymers were evaluated in this study. The stress-strain curve was depicted in Figure 6, and the mechanical property values derived from the stress-strain curve were listed in Table 2, with the exception of surface hardness and crosslinking density. The results clearly demonstrated that the mechanical properties of the prepared coating were significantly improved with the addition of copolymer under the same strain conditions. However, when the amount of copolymer added exceeded 16 g (P_16_ and P_20_), the stress-strain curve of the prepared coatings showed a certain degree of decline, and the measured 100% tensile stress and calculated elastic modulus also exhibited a significant decrease (although still higher than the control coating P_0_). In addition, the fracture elongation of the prepared coating was significantly enhanced with the incorporation of copolymers, with the fracture elongation of the P_20_ coating reaching as high as 200%, while that of the control coating P_0_ was only 120%. These results indicated that the incorporation of copolymers effectively enhanced the tensile fracture characteristics of the PDMS elastomer. The data also suggested that the addition of synthetic copolymer enhanced the coating’s mechanical properties, but excessive addition may result in a degradation of the coating’s mechanical properties under the same strain conditions.

To investigate the causes for the change in the mechanical properties of coatings, the crosslinking density of the samples was determined using the toluene swelling method, and specific testing requirements are described in other literature [18], where they are also cited in Supplementary File S5. To simplify, the influence of the copolymer on the Flory-Huggins interaction parameter between the prepared coating and toluene is disregarded, and the number-average molecular weight between adjacent crosslinking points Mc is used to represent the crosslinking density of the coating, where a higher value of Mc indicates a lower crosslinking density. The test results were also shown in Table 2. The results showed that with the addition of copolymers, the crosslinking density of the prepared coating increased, which improved its mechanical properties. The free copolymer contained within the coating fills the space of the three-dimensional crosslinked PDMS network structure. In order to store excess copolymers as the copolymer content increases, the three-dimensional crosslinked network must expand, resulting in incomplete crosslinking of the PDMS resin and a decrease in the crosslinking density of the coating, which decreases the coating’s elastic modulus and 100% tensile stress, respectively. The synthesized PTS copolymer used VMQ resin as the main chain structure, and VMQ resin is a common reinforcing polymer material. Therefore, with the increase in copolymer content, the fracture elongation of the prepared coating increased.

According to the testing procedure utilized in this investigation, the measured Shore hardness values tend to reflect the hardness of the coating surface region. Similar to the observation of the surface microstructure of the prepared coating (Figure 3), when the amount of copolymer added is small, due to the strong hydrogen bond interaction of the copolymer, the copolymer tends to accumulate in the coating-substrate region. Therefore, the copolymer content on the surface of the coating is lower, and the surface is flat and smooth, while the measured shore hardness value is comparable to that of the control coating. Adding more copolymers to the PDMS elastomer forces excess copolymers to be stored in the surface region of the coating, which not only degrades the surface smoothness of the prepared coating but also increases its surface hardness. Therefore, the measured Shore hardness value increases significantly.

### 2.4. The Leaching Behavior of PSO

**Figure 7:**
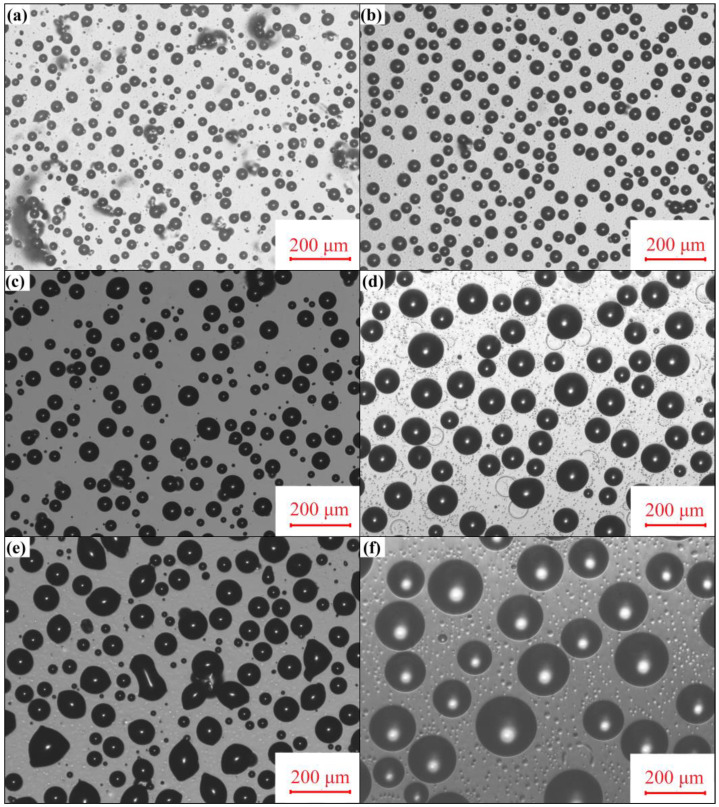
The leaching behavior of PSO on the surface after 30 days: (**a**) P_0_; (**b**) P_4_; (**c**) P_8_; (**d**) P_12_; (**e**) P_16_; and (**f**) P_20_.

**Figure 8:**
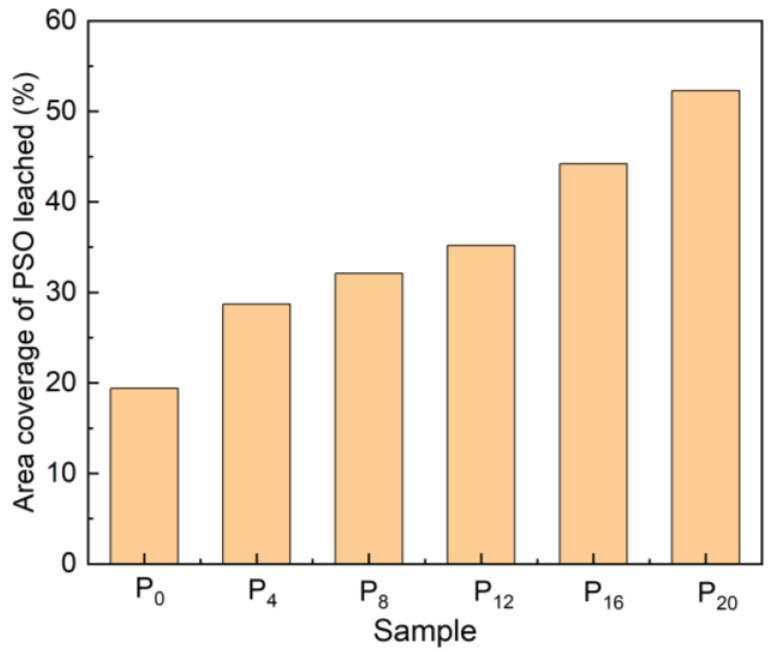
The area coverage of PSO on the surface.

The prepared coating surface was wiped with ethanol and then allowed to stand at room temperature in the air for 30 days to observe the leaching behavior of PSO on the coating surface (Figure 7). The coverage of the leached PSO on the coating surface was calculated using software (Figure 8). After PSO has been deposited on the coating surface, the droplets will aggregate, causing the size of the oil droplets to increase and their quantity to decrease. Observational data revealed that the amount of PSO leached increased in proportion to the quantity of copolymer added during the same exposure time. The previous research conclusion demonstrated that the increase in crosslinking density of the PDMS coating would inhibit the precipitation of PSO [17]. However, when the amount of copolymer added was less than or equal to 12 g, it could enhance the crosslinking density of the coating, but the PSO observed on the surface progressively increased at the same exposure time. Therefore, in this investigation, other factors exist that determine the leaching behavior of PSO.

**Figure 9:**
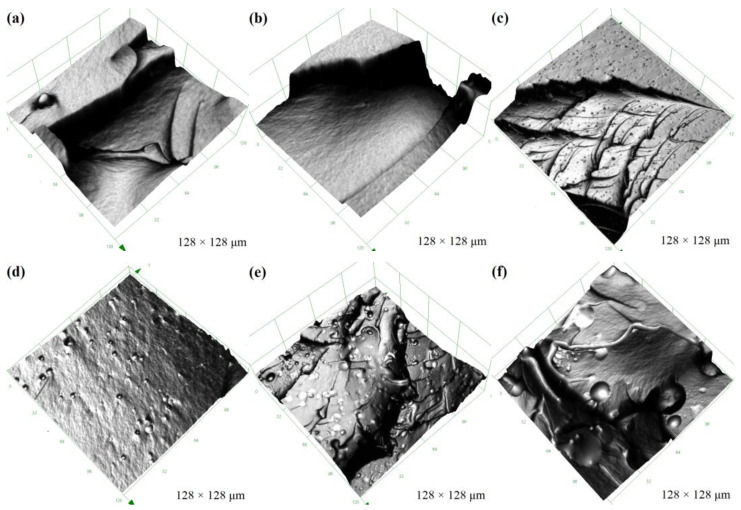
The fracture micro-morphologies of the prepared coatings, which were used to analyze the leaching behavior of PSO: (**a**) P_0_; (**b**) P_4_; (**c**) P_8_; (**d**) P_12_; (**e**) P_16_; and (**f**) P_20_.

After 30 days of exposure, the fracture morphology of a prepared coating was examined using CLSM equipment. The cast film was swollen with acetone before being tugged in a liquid nitrogen environment to obtain a non-deformed fracture. Previous research has demonstrated that acetone can dissolve free PSO. If the PSO exists within the coating as oil droplets, oil storage sacs can be observed through the above pretreatment. The Supplementary File S6 showed the fracture microstructure of the P_20_ coating observed directly without pretreatment, which also confirmed that PSO existed in the P_20_ coating in the form of oil storage sacs. The microscopic fracture morphology of all prepared coatings was examined (Figure 9). It was found that the PSO existed in different storage forms in the prepared coatings. Similar to the control coating P_0_, there were no oil storage sacs inside the prepared coating P_4_ with a small amount of copolymer addition, and the tissue structure of the prepared coating is uniform, with only tissue steps produced by stress concentration during the stretching process. With the increase in copolymer addition, oil storage sacs gradually appeared inside the coating. Similar to other studies, the number of oil storage sacs decreased and the volume increased within the same field of view, indicating an increase in the amount of PSO in the coating in the form of free oil droplets. The storage of PSO in the coating follows the theory of polymer solution, and the fact that PSO exists in the coating in the form of oil droplets indicates that PSO undergoes phase separation in the blended coating and is more likely to be leached from the coating surface. For P_0_ and P_4_ coatings, since the PSO was uniformly mixed in the PDMS elastomer and no phase separation occurred, it was more difficult for PSO to be leached from the coating surface. The molecular structure of the synthesized copolymer differed substantially from that of the PDMS resin and PSO, particularly the H-bonding interactions between its special functional groups, which were more likely to induce the non-polar PSO to undergo phase separation. With the increased copolymer addition, the tendency of the copolymer to drive the phase separation of PSO in the cured coating increased, and the leaching efficiency of PSO was further enhanced. Although the crosslinking density of the coating would limit the leaching behavior of PSO, the decisive factor for this study was the difference in molecular structure leading to the phase separation of PSO.

### 2.5. Bonding Strength between Coating and Substrate

The greatest obstacle to the commercial application of FR coatings based on PDMS is the poor binding performance between non-polar PDMS materials and polar substrates. In this work, a PTS copolymer was synthesized by introducing functional groups capable of forming H-bonding interactions in MQ-silicone resins, which in turn induced H-bonding adhesion effects in the blended coating to strengthen the adhesion between the coating and the substrate. The bonding strength between the prepared coating and the substrate was evaluated by pull-off testing (Table 3). Compared to the control coating P_0_, the pull-off strength of all prepared coatings with added copolymers was improved. When the amount of PTS added exceeded 16 g, the pull-off strength of the coating reached a plateau and no longer increased. The coating-substrate region of the prepared coating could not accommodate more copolymer, and excess copolymer was forced to accumulate in a region away from the substrate. Consequently, it was not possible to increase the adhesion strength between the coating and the substrate. However, excessive copolymers damaged the surface smoothness of the coating, and its influence on the anti-fouling performance needs to be evaluated.

### 2.6. Antifouling Performance

**Figure 10:**
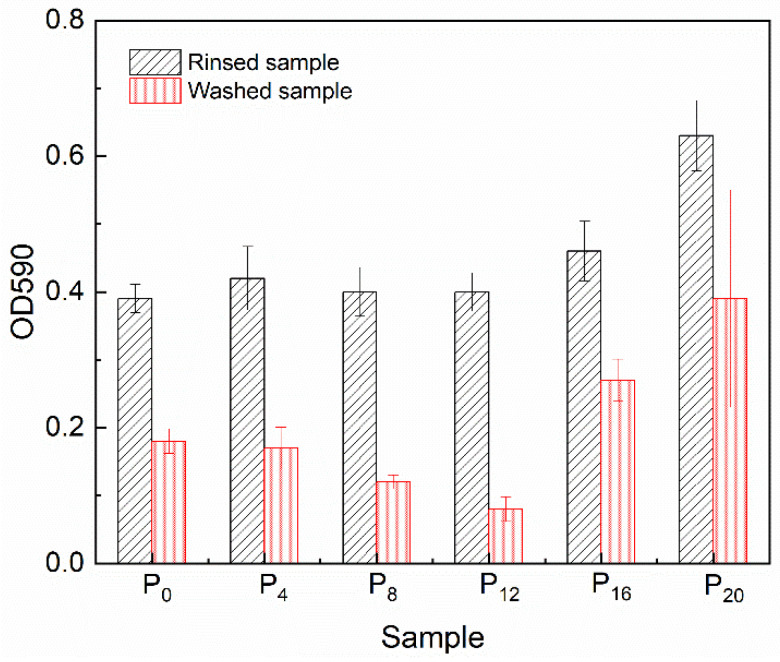
The marine bacterial biofilm assay.

The PSO that leached onto the coating surface for 30 days was cleaned with toluene, followed by the evaluation of the marine bacterial biofilm adhesion assay. Specifically, the coating antifouling evaluation test was conducted for 7 days, so the test samples were taken out and immersed in an equal amount of fresh seawater every 24 h. During the entire seawater replacement process, the sample was not treated in any way. The final evaluation results were shown in Figure 10. The incorporation of the synthetic copolymer into the prepared coatings significantly influenced their anti-fouling performance by changing the surface properties and mechanical properties. For the rinsed samples, the surface bacterial adhesion of P_4_, P_8_, and P_12_ was basically consistent with the control samples P_0_ (the value of OD590), although the increase in surface micro-roughness had enhanced the hydrophobicity of the prepared coating to some extent. Nevertheless, the surface free energy of the pertinent coating was within the optimal range, so its influence on anti-fouling performance was minimal. With further increases in the copolymer content, the bacterial biofilm adhered to the rinsed sample surface increased significantly (the OD590 value increased). Although the surface free energy of the P_16_ and P_20_ samples increased, they were still within the range of effective anti-fouling. Therefore, the core reason for the decline in the anti-fouling performance of the rinsed coating was the deterioration of the coating surface flatness. The rough surface provided a foothold for marine organisms to adhere to, making it easier to adhere to the surface.

Regarding FR antifouling coatings, the coating’s elastic modulus determines the re-release of adhered marine organisms. A high elastic modulus can make it more challenging to re-detach attached organisms. The fouling-release performance of prepared coatings was evaluated by washing the sample. Although the addition of copolymers led to a significant increase in the elastic modulus of the coating, which reached a maximum value on the P_12_ coating, testing the washed samples revealed that the P_12_ sample had the smallest residual adhering fouling organisms after standard seawater flow washing. The leaching of PSO onto the surface could significantly improve the antifouling effect of the coating and, more importantly, could wrap and remove attached fouling organisms [19,22,27]. For the prepared coating, the incorporation of copolymers ensured excellent PSO leaching behavior, which could compensate for the decrease in the fouling-release capability of the coatings due to the increase in the elastic modulus. Although the P_16_ and P_20_ coating surfaces had higher PSO leaching efficiency, excessively leached PSO could only lead to waste and a reduction in the leaching lifespan and could not further improve the fouling-release ability of the coatings. In addition, a too-rough surface increased the adhesion of marine bacteria, and the attached biofilm was denser, making it more difficult to remove attached bacteria using PSO. The evaluation of marine bacterial biofilm adhesion on the prepared coatings indicated that the P_12_ coating had the best antifouling performance.

## 3. Materials and Methods

### 3.1. Materials

The 2-(2-ethoxyethoxy)ethyl acrylate (EOEOEA) and 1-allyl-2-thiourea (ATU) were acquired from Aladdin (Shanghai, China). Sinopharm Chemical Reagent Co., Ltd. (Shanghai, China) supplied 2,2′-azobis(2-methylpropionitrile) (AIBN). Hydroxyl-terminated polydimethylsiloxane (PDMS) with a viscosity of 10,000 mPa∙s and methyl-vinyl MQ silicone resin (VMQ) with a vinyl group concentration of 0.08 mol/100 g were procured from Dayi Chemical Industry Co., Ltd. (Yantai, China), while the molar ratio of VMQ structural units is 1.4:1 (M:Q). Phenylmethylsilicone oil (PSO) with a viscosity of 30 mPa∙s was purchased from Xinda Chemical Co., Ltd. (Bengbu, China). Tetraethylorthosilicate (TEOS), toluene, xylene, acetone, acetylacetonate, and ethanol were acquired from Kemiou Chemical Reagent Company (Tianjin, China). The above chemicals were chemically pure. Bismuth neodecanoate (BiND) was obtained from Deyin Chemical Co., Ltd. (Shanghai, China). All reagents were utilized without any additional purification. In addition, laboratory-made deionized water was employed for this investigation.

### 3.2. Synthesis of Polymers

The PTS was synthesized by the bulk free-radical copolymerization of ATU, EOEOEA, and VMQ in the absence of a solvent. In a four-necked round-bottom flask equipped with a reflux apparatus, the specified amounts of monomers and AIBN were combined and stirred to form a homogenous solution. The AIBN content was maintained at 1 wt.% (relative to the total monomers). The entire reaction was carried out at 55 °C for 4 h in an atmosphere of nitrogen. The synthesized copolymer was then submerged in a large volume of deionized water to remove residuals and contaminants. Next, the copolymer was extracted at least three times with acetone. After precipitation and drying, the copolymer was ultimately stored in equal-weight alcohol. Based on the miscibility of two monomers within a specific percentage range, the molar ratio of ATU and EOEOEA was maintained at 0.85:0.15. Furthermore, 6.5 mol% of VMQ was present in total monomers.

### 3.3. Preparation of Coatings

The PDMS-based FR coating consists of three components: the pre-dispersed slurry (Part A), which is comprised of PDMS, PTS, and PSO; the curing agent (Part B), which refers to the xylene solution of TEOS; and the catalytic agent (Part C), which is the acetylacetonate solution of BiND. A two-step procedure was devised for coating preparation. Using a 500 mL stirring tank, the copolymer-alcohol mixture (20 g) was reblended uniformly and then mixed into the PDMS resin (100 g). After 15 min of mixing at 800 rpm, PSO (5 g) was added to the stirring tank and stirred for more than 30 min. Next, three components were combined uniformly based on the following mass ratio: PDMS: Part B: Part C = 20:4:1. The substrates consisted of tin plates (150 mm × 50 mm × 0.5 mm) and Teflon molds (150 mm × 150 mm × 5 mm). To regulate the coating thicknesses on the tin plates, a 200-µm-thick BGD201 wet film preparer (BIUGED laboratory instrument, Guangzhou, China) was utilized. In addition, the cast films were cured by applying the coatings to the Teflon molds’ grooves. To obtain the cured samples, they were cured under vacuum at 25 °C for more than 24 h. The mechanical and morphological features of the cast films were evaluated, while the surface properties and antifouling performance of the prepared coatings were evaluated. Specifically, the coating thickness on the tin plates required to be maintained between 200 and 250 µm. The acetone swelling procedure was used to detach the cast films that had stuck to the Teflon molds. Before conducting tests, the films peeled from Teflon molds had to be dried (at 25 °C for 48 h in a nitrogen environment).

After preparation, all tests were carried out at 25 °C. The prepared samples are expressed as P_x_, where x denotes the addition amount of PTS, and P_0_ represents the control sample without the copolymer.

### 3.4. Characterization of Polymers

With a scan range of 4000–650 cm^−1^ and a resolution of 2 cm^−1^, a Spotlight 200i Fourier transform infrared (FT-IR) spectrophotometer (PerkinElmer Enterprise Management Co., Ltd., Shanghai, China) was utilized for measurements. Thirty-two scans were obtained for each sample. The KBr disk technique was adopted to evaluate the FT-IR spectra of copolymers and monomers. 

^1^H nuclear magnetic resonance (^1^H-NMR) spectroscopy was recorded on a Bruker AVANCE III HD spectrometer (400 MHz, Bruker, Karlsruhe, Germany) in CDCl_3_.

The molecular weight and dispersion of the copolymers were determined utilizing PL-50 normal-temperature gel permeation chromatography (GPC; POLYTECH Co., Ltd., Beijing, China). The mobile phase consisted of 1 mL/min of tetrahydrofuran.

### 3.5. Surface Properties

A JC2000C contact test system was used to conduct static contact angle testing (Zhongchen Co., Ltd., Shanghai, China). Using a syringe, 3-L droplets of deionized H_2_O and CH_2_I_2_ were sprayed onto the coated surface. Digital images of droplet silhouettes were captured using a charge-coupled device camera. Then, the contact angles were determined using the measuring angle method. Each specimen was evaluated at six distinct sites. The two-liquid approach of Owens was used to calculate the SFE values [28]. The coatings were cleaned with alcohol prior to the measurements.

Using confocal laser scanning microscopy (CLSM, OLS4000, Olympus (China) Co., Ltd., Beijing, China) with different fields of view, the surface microstructure of prepared coatings was analyzed. Meanwhile, the surface roughness (Sa) values were also examined with LEXT software (version 2.2.4).

### 3.6. Mechanical Properties

In compliance with GB/T 528-1998, a WDW-5 auto tensile tester (Xinbiao Automation Equipment Manufacturing Co., Jinan, China) with a crosshead speed of 30 mm/min was used to measure tensile strength. For each manufactured coating, three samples were tested, and elastic moduli were determined using stress–strain data for strains less than 0.2 mm/mm. The stress–strain curve was built by collecting data close to the mean elastic modulus values [29].

Using an HT220 shore hardness tester, the samples’ hardness was evaluated (Time High Technology Co., Ltd., Beijing, China). The test standards permit the use of coatings with a thickness greater than 2 mm. Thus, cast films with thicknesses above 4 mm were used.

### 3.7. Coating Structure

CLSM was also used to measure the fractured microstructure of prepared coatings. The following procedures were performed on the samples prior to observation: the films obtained by the acetone swelling method were shattered in a liquid nitrogen atmosphere using tensile testing equipment (FULETEST Co., Ltd., Shanghai, China). After 15 min at room temperature, the shattered films could be tested.

The elemental composition of prepared coatings from different regions was investigated using X-ray photoelectron spectroscopy (XPS, Thermo VG, Waltham, MA, USA). Cutting the cast film samples to obtain the structure of the surface region (coating-air) and then conducting a high-temperature embrittlement treatment (80 °C for 24 h in a nitrogen environment). Subsequently, XPS tests were carried out according to the process.

### 3.8. Bonding Strength between Coating and Substrate

For measuring the pull-off strength in accordance with BG/T 5210-85, a BGD500 portable adhesion tester (BIUGED laboratory instrument, Guangzhou, China) with an accuracy of 0.01 MPa was utilized. Due to the low SFE, the inability of the prepared coating to attach to the binder forced the development of a more effective test method; for which the complete testing methodology is disclosed elsewhere [29]. The pull-off strength of the copolymer was additionally tested. 

### 3.9. PSO Leaching Behavior

Using a DYE-400E polarized light microscope with a hot stage (Dian Ying Optics, Shanghai, China) at 25 °C, PSO leached onto the prepared coated surface throughout the exposure duration. Moreover, the storage behavior of PSO inside the coatings could be determined by the fracture morphology measure. The processing of PSO leaching behavior images was conducted using the Image-Pro Plus software (version 5.1). All images obtained were first processed by grayscale mapping and then by the two-value operation. Then images were evaluated using statistical capabilities.

### 3.10. Coating Antifouling

Biofilm adhesion assays were conducted to analyze the antifouling effectiveness of prepared coatings. The biofilm attached to the surface of the coating was collected using crystal violet staining, the test process for which is described elsewhere [15,18,30]. It was characterized by analyzing the absorbance of the supernatant at 590 nm by using an ultraviolet-visible spectrophotometer (NYSE: A, Palo Alto, CA, USA). The adhering biofilm removal rate might therefore be computed using Formula (1), where *D_a_* denotes the absorbance of the washed samples, *D_b_* denotes the absorbance of the rinsed samples, and R denotes the removal rate of the adhered biofilm.

(1) $$R=\frac{D_{b}-D_{a}}{D_{b}}\times 100\%$$

Before concluding the biofilm adhesion experiment, the prepared coating must be applied over the whole surface of the tin plate. This can avoid corrosion of the substrate and provide accurate test results.

## 4. Conclusions

This work synthesized a PTS copolymer through free radical polymerization by incorporating thiourea and ether groups onto the MQ silicone polymer. The molecular structure of the synthesized copolymer was characterized by FT-IR, ^1^H-NMR, and GPC, which exhibited strong H-bonding interactions and a narrow molecular weight polydispersity index. Based on the copolymer, the FR coatings were prepared. The incorporation of copolymers increased the micro-roughness of the coating surface, which enhanced its hydrophobicity to some extent, but excessive copolymers led to the deterioration of surface smoothness, resulting in a hydrophilic surface. XPS analysis showed that the excess copolymer forced the surface area of the coating to accommodate it, resulting in surface morphology degradation and increased surface roughness due to differences in the molecular structure of the copolymer and PDMS elastomers. The copolymer stored in its free state in the gaps of the three-dimensional crosslinked network effectively improved the mechanical properties of the coating. However, excessive copolymers reduced the crosslinking density of the coating, leading to a decrease in its mechanical properties, although the coating’s fracture elongation increased with the addition of copolymers. Shore hardness measurement showed excessive copolymer was concentrated in the surface region of the prepared coatings, resulting in increased surface region hardness. The copolymer promoted the leaching of PSO onto the coating surface. In-depth research revealed that copolymers changed the storage form of PSO inside the coating, and with increasing copolymer addition, the trend of copolymers driving PSO to phase separate in the cured coating increased and the leaching efficiency of PSO was further improved. The copolymer’s H-bonding interactions substantially strengthened the adhesion between the coating and the substrate. However, the amount of copolymers that can be stored in the interface region was limited, and excessive copolymer addition did not further improve the bonding strength between the coating and the substrate. The antifouling performance test showed that an appropriate amount of copolymer could effectively improve the antifouling performance of the coating. Among the coatings prepared in this study, P_12_ showed the best anti-fouling performance.

## Figures and Tables

**Table 1:** The surface properties of the prepared coatings.

Sample	Contact Angle (°)		Surface Free Energy (mJ/m^2^)
	Water	Diiodomethane	
P_0_	108.6 ± 0.32	68.9 ± 0.65	23.7
P_4_	108.5 ± 0.63	69.5 ± 0.66	23.4
P_8_	109.8 ± 0.55	70.3 ± 0.47	22.7
P_12_	111.4 ± 0.31	72.5 ± 0.38	21.8
P_16_	96.1 ± 0.65	67.6 ± 0.31	24.7
P_20_	88.6 ± 1.12	61.1 ± 1.03	29.1

**Table 2:** The mechanical properties of the prepared coatings (±SEM, *n* = 3).

Sample	Elastic Modulus (MPa)	100% Tensile Stress (MPa)	Breaking Elongation (%)	Shore Hardness (HA)	Crosslinking Density (Mc)
P_0_	0.31 ± 0.027	0.251	100.7	12.8 ± 0.55	19,840 ± 374
P_4_	0.61 ± 0.030	0.290	123.2	13.3 ± 0.31	14,533 ± 536
P_8_	0.78 ± 0.042	0.345	138.8	14.6 ± 0.67	12,011 ± 219
P_12_	0.81 ± 0.102	0.414	165.7	16.7 ± 1.05	9870 ± 343
P_16_	0.57 ± 0.061	0.278	197.4	23.5 ± 0.75	15,611 ± 406
P_20_	0.50 ± 0.038	0.256	259.1	26.7 ± 0.80	17,520 ± 277

**Table 3:** The pull-off strength of the prepared coatings.

Sample	P_0_	P_4_	P_8_	P_12_	P_16_	P_20_
Pull-off strength (MPa)	0.08 ± 0.011	0.15 ± 0.027	0.24 ± 0.018	0.35 ± 0.037	0.41 ± 0.031	0.40 ± 0.040

## Data Availability

The data are contained within the article.

## Supplementary Materials

The following supporting information can be downloaded at: https://www.mdpi.com/article/10.3390/molecules28083574/s1, Supplementary File S1: The GPC measurement of polymer; Supplementary File S2: Detailed description of ^1^H-NMR; Supplementary File S3: The ^1^H-NMR spectra of VMQ; Supplementary File S4: The FT-IR spectra of the monomers; Supplementary File S5: The method for the calculation of crosslinking density; Supplementary File S6: The fracture micro-morphology of the prepared coating P_20_ without any treatment.

## References

[1] Xiong G., Zhang Z., Qi Y. (2022). Effect of the properties of long afterglow phosphors on the antifouling performance of silicone fouling-release coating. Prog. Org. Coat..

[2] Guo H., Liu X., Zhao W., Xie C., Zhu Y., Wen C., Li Q., Sui X., Yang J., Zhang L. (2021). A polyvinylpyrrolidone-based surface-active copolymer for an effective marine antifouling coating. Prog. Org. Coat..

[3] Wu T., Qi Y., Chen Q.A., Gu C., Zhang Z. (2022). Preparation and Properties of Fluorosilicone Fouling-Release Coatings. Polymers.

[4] Tian L., Yin Y., Bing W., Jin E. (2021). Antifouling Technology Trends in Marine Environmental Protection. J. Bionic Eng..

[5] Han X., Wu J., Zhang X., Shi J., Wei J., Yang Y., Wu B., Feng Y. (2021). The progress on antifouling organic coating: From biocide to biomimetic surface. J. Mater. Sci. Technol..

[6] Ferreira O., Rijo P., Gomes J.F., Santos R., Monteiro S., Vilas-Boas C., Correia-da-Silva M., Almada S., Alves L.G., Bordado J.C. (2020). Biofouling Inhibition with Grafted Econea Biocide: Toward a Nonreleasing Eco-Friendly Multiresistant Antifouling Coating. ACS Sustain. Chem. Eng..

[7] Chen Q.A., Zhang Z., Qi Y. (2022). Influence of different silicone oils on properties of MWCNTs-OH/PDMS coatings. Surf. Eng..

[8] Kolle S., Ahanotu O., Meeks A., Stafslien S., Kreder M., Vanderwal L., Cohen L., Waltz G., Lim C.S., Slocum D. (2022). On the mechanism of marine fouling-prevention performance of oil-containing silicone elastomers. Sci. Rep..

[9] Qiu H., Gapeeva A., Hoelken I., Kaps S., Adelung R., Baum M.J. (2022). Preventing algae adhesion using lubricant-modified polydimethylsiloxane/polythiourethane nanocomposite. Mater. Des..

[10] Chen J., Zhao J., Lin F., Zheng X., Jian R., Lin Y., Wei F., Lin Q., Bai W., Xu Y. (2023). An FFT-based method for estimating the in-plane elastic properties of honeycomb considering geometric imperfections at large elastic deformation. Prog. Org. Coat..

[11] Gu Y., Yu L., Mou J., Wu D., Xu M., Zhou P., Ren Y. (2020). Research Strategies to Develop Environmentally Friendly Marine Antifouling Coatings. Mar. Drugs.

[12] Guazzelli E., Perondi F., Criscitiello F., Pretti C., Oliva M., Casu V., Maniero F., Gazzera L., Galli G., Martinelli E. (2020). New amphiphilic copolymers for PDMS-based nanocomposite films with long-term marine antifouling performance. J. Mater. Chem. B.

[13] Eduok U., Faye O., Szpunar J. (2017). Recent developments and applications of protective silicone coatings: A review of PDMS functional materials. Prog. Org. Coat..

[14] Hu P., Xie Q., Ma C., Zhang G. (2021). Fouling resistant silicone coating with self-healing induced by metal coordination. Chem. Eng. J..

[15] Hu P., Xie Q., Ma C., Zhang G. (2020). Silicone-Based Fouling-Release Coatings for Marine Antifouling. Langmuir.

[16] Yang M., Sun Y., Chen G., Wang G., Lin S., Sun Z. (2020). Preparation of a self-healing silicone coating for inhibiting adhesion of benthic diatoms. Mater. Lett..

[17] Ba M., Zhang Z.-P., Qi Y.-H. (2018). The leaching behavior of phenylmethylsilicone oil and antifouling performance in nano-zinc oxide reinforced phenylmethylsilicone oil-Polydimethylsiloxane blend coating. Prog. Org. Coat..

[18] Ba M., Zhang Z., Qi Y. (2018). Fouling Release Coatings Based on Polydimethylsiloxane with the Incorporation of Phenylmethylsilicone Oil. Coatings.

[19] Shivapooja P., Cao C., Orihuela B., Levering V., Zhao X., Rittschof D., Lopez G.P. (2016). Incorporation of silicone oil into elastomers enhances barnacle detachment by active surface strain. Biofouling.

[20] Yuan H., Hao R., Sun H., Zeng W., Lin J., Lu S., Yu M., Lin S., Li J., Chen L. (2022). Engineered Janus cellulose membrane with the asymmetric-pore structure for the superhigh-water flux desalination. Carbohydr. Polym..

[21] Sun H., Liu Z., Liu K., Gibril M.E., Kong F., Wang S. (2021). Lignin-based superhydrophobic melamine resin sponges and their application in oil/water separation. Ind. Crops Prod..

[22] Yang Q., Zhang Z., Qi Y., Zhang H. (2021). The Antifouling and Drag-Reduction Performance of Alumina Reinforced Polydimethylsiloxane Coatings Containing Phenylmethylsilicone Oil. Polymers.

[23] Selim M.S., Elmarakbi A., Azzam A.M., Shenashen M.A., El-Saeed A.M., El-Safty S.A. (2018). Eco-friendly design of superhydrophobic nano-magnetite/silicone composites for marine foul-release paints. Prog. Org. Coat..

[24] Yang W.J., Neoh K.-G., Kang E.-T., Teo S.L.-M., Rittschof D. (2014). Polymer brush coatings for combating marine biofouling. Prog. Polym. Sci..

[25] Xue J., Wang L., Fan Y., Xu J., Zhao J., Tian L., Du W. (2022). Mechanically Enhanced Self-Stratified Acrylic/Silicone Antifouling Coatings. Coatings.

[26] Wang Y.-J., He Y., Zheng S.Y., Xu Z., Li J., Zhao Y., Chen L., Liu W. (2021). Polymer Pressure-Sensitive Adhesive with A Temperature-Insensitive Loss Factor Operating Under Water and Oil. Adv. Funct. Mater..

[27] Galhenage T.P., Hoffman D., Silbert S.D., Stafslien S.J., Daniels J., Miljkovic T., Finlay J.A., Franco S.C., Clare A.S., Nedved B.T. (2016). Fouling-Release Performance of Silicone Oil-Modified Siloxane-Polyurethane Coatings. ACS Appl. Mater. Interfaces.

[28] Owens D.K., Wendt R.C. (1969). Estimation of the surface free energy of polymer. J. Appl. Polym. Sci..

[29] Zhou H., Zheng Y., Ba M., Kong J., Wang Y. (2021). Self-stratified fouling release coatings based on polydimethylsiloxane incorporated with acrylate-MQ silicone copolymer. Prog. Org. Coat..

[30] Fan F., Zheng Y., Ba M., Wang Y., Kong J., Liu J., Wu Q. (2021). Long time super-hydrophobic fouling release coating with the incorporation of lubricant. Prog. Org. Coat..

