# Supplementary material for: Polyether-Thiourea-Siloxane Copolymer Based on H-Bonding Interaction for Marine Antifouling
Source: Molecules. 2023 Apr 19;28(8):3574. doi: 10.3390/molecules28083574 (PMC10144924; doi:10.3390/molecules28083574)

Supplementary File S1. The GPC measurement of polymer.

The GPC spectra of the copolymer PTS. The test accuracy of this equipment is between 300 and 2, 600, 000.

Cirrus GPC Sample Injection Report

Generated by: LY02 Thursday, July 08, 2021 2:36 AM

Workbook: D:\Cirrus Workbooks\20210518\20210518.plw

Sample Details

Sample Name: Zhang

Acquired: 7/8/2021 2:04:49 AM By Analyst: LY02 Batch Name: 7_7_2021

Concentration: 0.10 mg/ml Injection Volume: 100.0 ul K of Sample: 14.1000 Alpha of Sample: 0.7000

Analysis Using Method: 20210604

Calibration Used: 6/21/2021 1:06:47 AM

Calibration Type: Narrow Standard Curve Fit Used: 1 K: 14.1000 Alpha: 0.7000

Calibration Curve: y = 10.354906 - 0.443666x^1

High Limit MW RT: 9.70 mins Low Limit MW RT: 18.25 mins

Flow Marker RT: 0.00 mins FRCF: 1.0000 FRM Name:

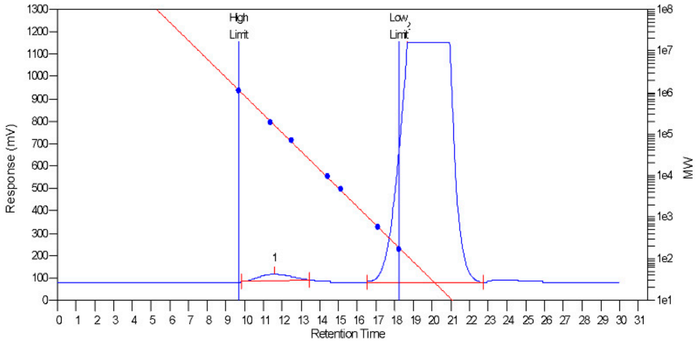

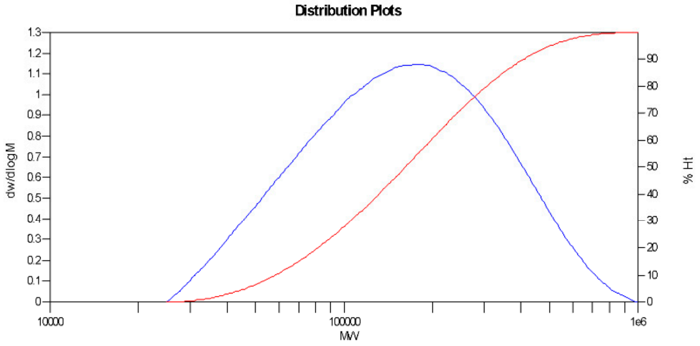

MW Averages

Peak No	Mp	Mn	Mw	Mz	Mz+1	Mv	PD
1	182027	120718	200874	306201	410702	186993	1.66399
2	11	25	68	153	254	59	2.72

Processed Peaks

Peak No	Name	Start RT (mins)	Max RT (mins)	End RT (mins)	Pk Height (mV)	% Height	Area (mV.secs)	% Area
1		9.82	11.62	13.43	30.2006	2.73628	3578.7	1.70538
2		16.55	18.68	22.75	1073.51	97.2637	206269	98.2946

The GPC spectra of the copolymer VMQ.

Cirrus GPC Sample Injection Report

Generated by: LY02 Wednesday, July 07, 2021 10:56 PM
Workbook: D:\Cirrus Workbooks\20210518\20210518.plw

Sample Details
Sample Name: YiXIJIMQ
Acquired: 7/7/2021 10:22:28 PM By Analyst: LY02 Batch Name: x
Concentration: 0.10 mg/ml Injection Volume: 100.0 ul K of Sample: 14.1000 Alpha of Sample: 0.7000
Analysis Using Method: 20210604

Calibration Used: 6/21/2021 1:06:47 AM
Calibration Type: Narrow Standard Curve Fit Used: 1 K: 14.1000 Alpha: 0.7000
Calibration Curve: y = 10.354906 - 0.443666x^1
High Limit MW RT: 9.70 mins Low Limit MW RT: 18.25 mins
Flow Marker RT: 0.00 mins FRCF: 1.0000 FRM Name:

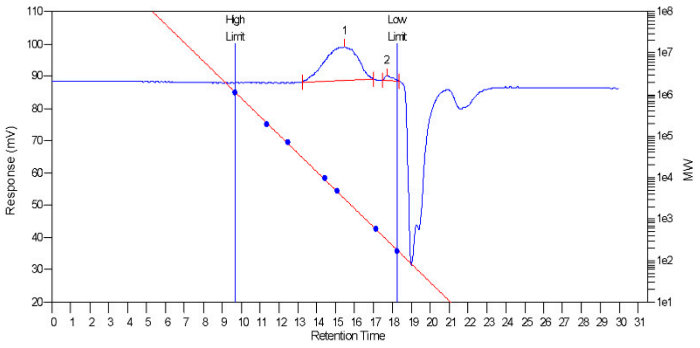

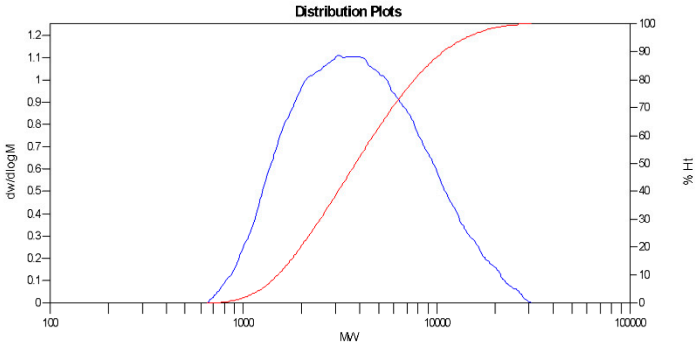

MW Averages

Peak No	Mp	Mn	Mw	Mz	Mz+1	Mv	PD
1	3164	3007	5145	8628	12581	4732	1.71101
2	312	265	276	285	294	274	1.04151

Processed Peaks

Peak No	Name	Start RT (mins)	Max RT (mins)	End RT (mins)	Pk Height (mV)	% Height	Area (mV.secs)	% Area
1		13.22	15.45	17.00	10.3366	87.5776	1260.89	96.8177
2		17.47	17.72	18.35	1.4662	12.4224	41.4438	3.18227

Supplementary File S2. Detailed description of 1H-NMR.

Detailed description of ^{1}H-NMR (PTS)

Mark	Chemical shift	Description
Ha	0.136	-Si-CH_3
Hb	0.952	-CH_3
Hc	1.20	-CH_2-Si-
Hd	1.37	-CH_3
He	1.60	H_2O
Hf	1.77	-CH-
Hj	3.52~3.63	-CH_2-CH_2-
Hh	4.32	-CO-O-CH_2-
Hi	9.01	-NH_2
Hg	9.53	-NH-

Supplementary File S3. The 1H-NMR spectra of VMQ.

The ^{1}H-NMR spectra of VMQ

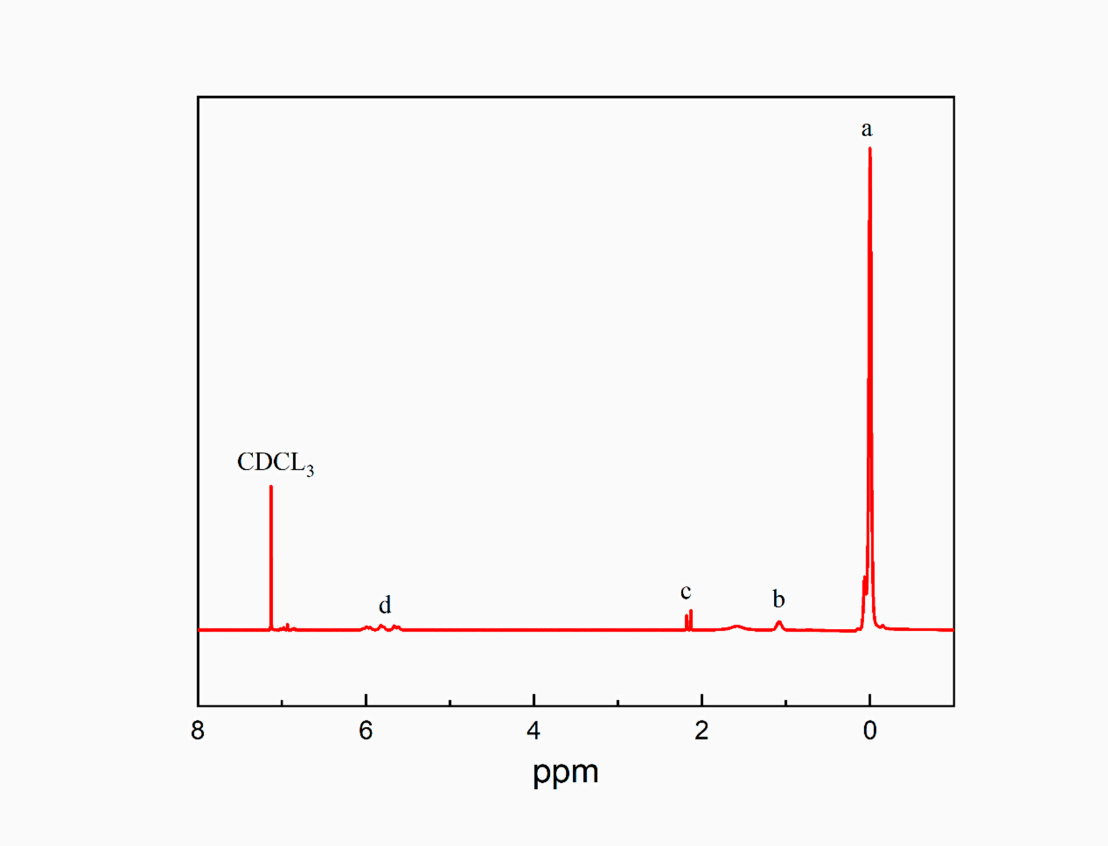

Detailed description of ^{1}H-NMR (VMQ): a, b, c, d represent the chemical shift appearing in the ^{1}H-NMR.

Mark	Chemical shift	Description
Ha	0.136	-Si-CH_3
Hb	1.10	-CH_2-Si-
Hc	2.18	-C=C-CH_3
Hd	5.58-5.98	–CH=C-

Supplementary File S4. The FT-IR spectra of the monomers.

The FT-IR spectra of the monomers: (a) EOEOEA; (b) ATU; (c) VMQ

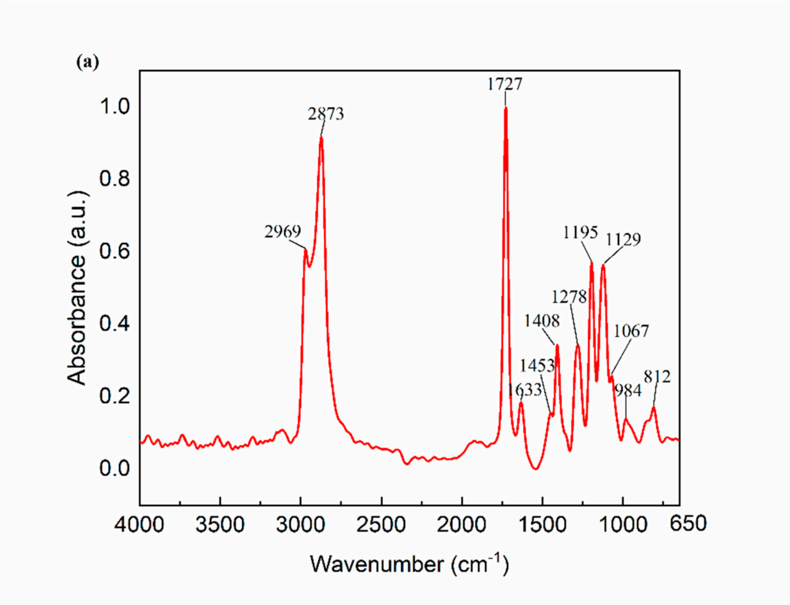

The olefinic groups in the spectra of EOEOEA at 1633 cm^{-1} and 984 cm^{-1}

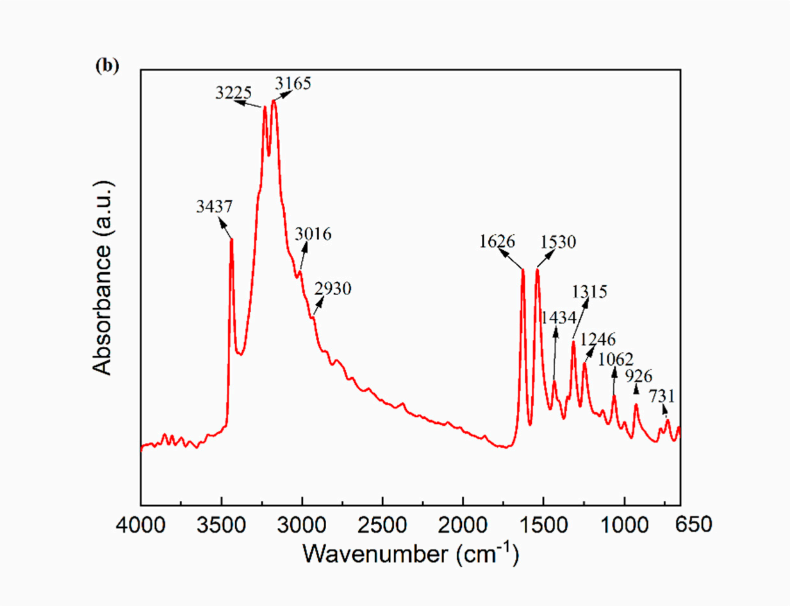

The olefinic groups in the spectra of ATU at 3016 cm^{-1} and 1626 cm^{-1}

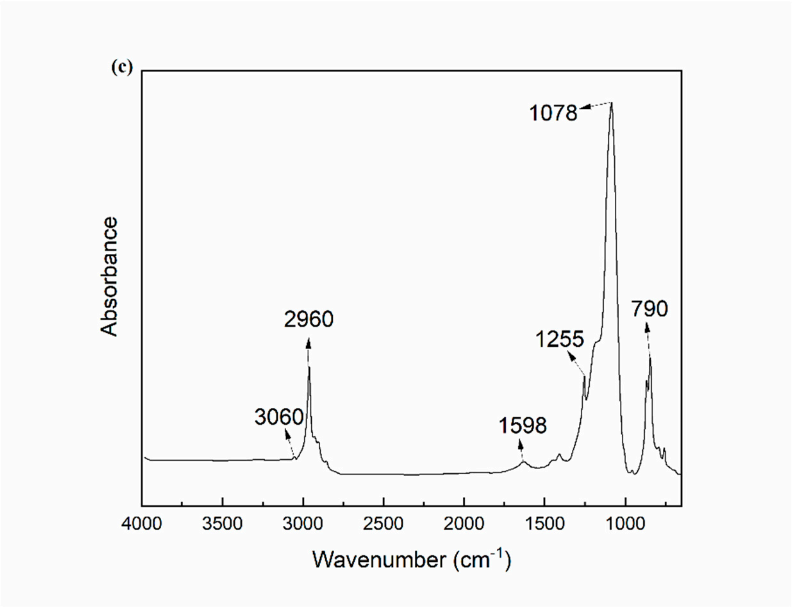

The olefinic groups in the spectra of VMQ at 3060 cm^{-1} and 1598 cm^{-1}

Supplementary File S5. The method for the calculation of crosslinking density.

The method for the calculation of crosslinking density, which were listed in the reference (Miao Ba, et al. Coatings, 2018, 153)

In order to figure out the origin of the mechanical properties of the coating, the crosslink density of the PDMS/PSO-blend membrane was measured by the equilibrium swollen method via using toluene as the solvent.[15]. Generally, it can be expressed by the molecular weight between crosslink points (Mc). Toluene can dissolve the PSO in the coating sample, and then the swelling of the gel can be determined. The value of Mc was calculated by the Flory-Rhener relation,

$$\mathrm{M}_C = \frac{-\rho V(v^{\frac{1}{3}} - v/2)}{\ln(1-v) + v + \chi_1 * v^2} \quad \text{(S1)}$$

Where v represents the volume fraction of the polymer in the swollen specimen [15], ρ represents the density of samples before swelling, V represents the molar volume of solvent and it is 106.125 cm3/mol for toluene, χ1 refers to the Flory-Huggins interaction parameter of samples and toluene, and it is 0.45 in this experiment.

In order to ensure the accuracy of experimental data, the weight of the samples was measured every 3 h by using a precision balance purchased from Mettler Toledo Co., Ltd. (Zurich, Switzerland) during the swelling process. When the difference of adjacent measurement data did not exceed 0.1 mg, the sample was up to the equilibrium swelling state. And the whole experiment was performed at 25 oC, while three samples for each coating were used in experiments.

Supplementary File S6. The fracture micro-morphology of the prepared coating P20 without any treatment.

The fracture micro-morphology of the prepared coating P_{20} was obtained without any treatment. The obvious phenomenon of PSO accumulation and storage was observed.

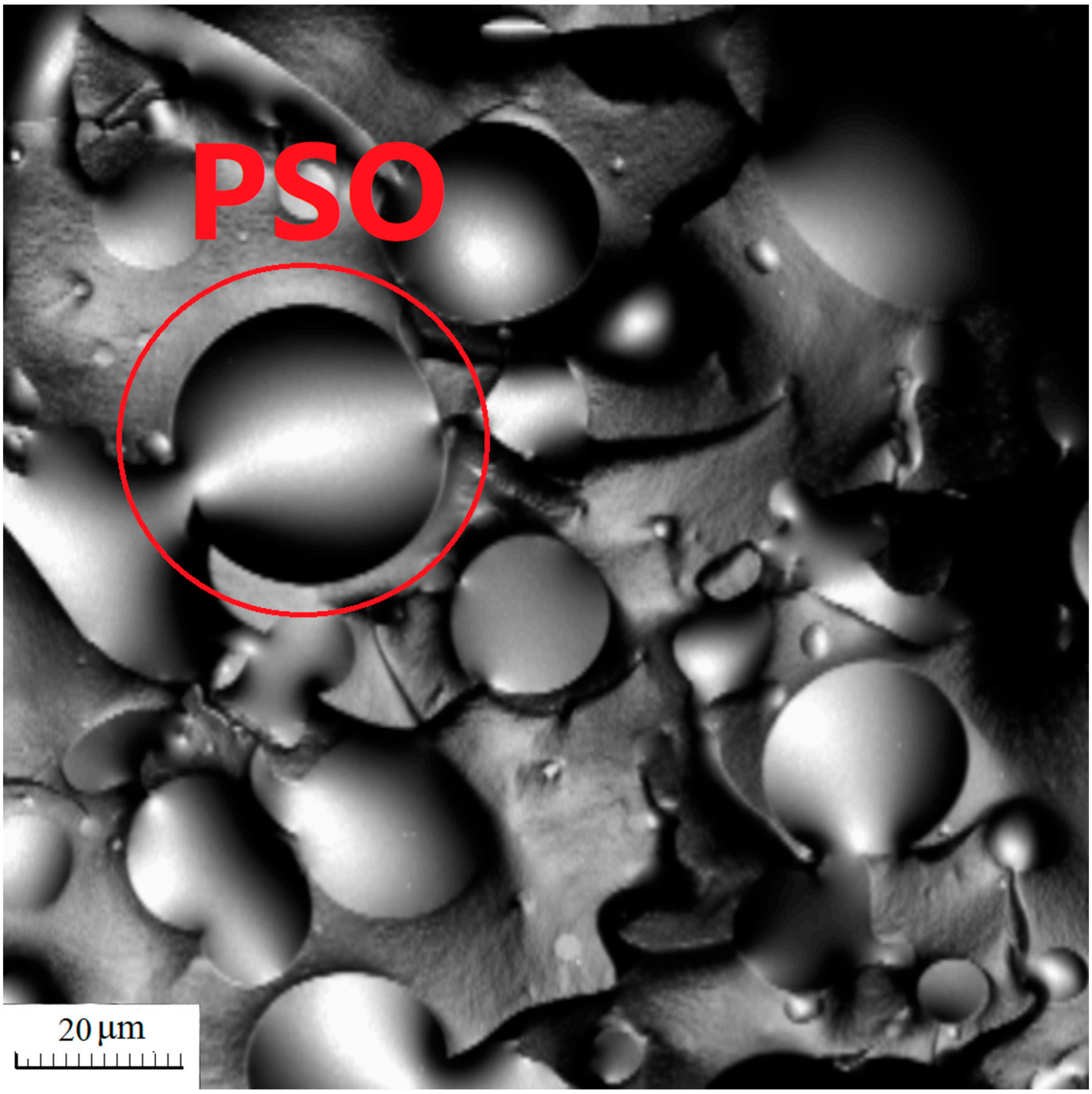

Supplement: Supplementary file 1 [file molecules-28-03574-s001.zip › molecules-2332332-supplementary.pdf]
